# Evaluating the epidemiology of vaginitis in a contemporary cohort: a population-based study

**DOI:** 10.3389/fpubh.2024.1486356

**Published:** 2025-02-25

**Authors:** Deirdre Church, Christopher Naugler, Maggie Guo, Ranjani Somayaji

**Affiliations:** ^1^Department of Pathology & Laboratory Medicine, Cumming School of Medicine, University of Calgary, Calgary, AB, Canada; ^2^Department of Medicine, Cumming School of Medicine, University of Calgary, Calgary, AB, Canada; ^3^Alberta Precision Laboratories, Alberta Health Services, Calgary, AB, Canada; ^4^Department of Family Medicine, Cumming School of Medicine, University of Calgary, Calgary, AB, Canada; ^5^Department of Community Health Sciences, Cumming School of Medicine, University of Calgary, Calgary, AB, Canada; ^6^Department of Microbiology, Immunology & Infectious Disease, Cumming School of Medicine, University of Calgary, Calgary, AB, Canada

**Keywords:** vaginitis, epidemiology, population-based, bacterial vaginosis, trichomoniasis, vulvovaginal candidiasis

## Abstract

**Introduction:**

Bacterial vaginosis (BV), vulvovaginal candidiasis (VVC), and *Trichomonas vaginalis* (TV) commonly cause infectious vaginitis in women, especially those of reproductive age. Characterization of the epidemiology of infectious vaginitis in a contemporary population-based cohort was done to determine the longitudinal age-standardized and sex-based testing, positivity, and incidence rates and ratios of infectious vaginitis in a large Canadian healthcare region.

**Methods:**

We conducted a population-based cohort study from March 2015 through March 2018 using the Alberta Precision Laboratories (APL) microbiology database. Calgary 2016 census data was used to calculate incidence rates (IR) and ratios (IRR) for cases and testing rates.

**Results:**

For testing and positivity, female sex, and younger age groups were associated with increased risk of BV, VVC, and TV infections. The annual mean population in 2016 was 1,411,660 individuals (50.3% female). A total of 40,259 cases/293,853 tests (13.7%) of BV, 32,894 cases/293,853 tests (11.2%) of VVC, and 2018 cases/342,986 tests (0.7%) of TV were identified. The overall IR for BV ranged from 0 to 609 cases per 10,000 person-years. The overall IR for VVC ranged from 0 to 445 per 10,000 person-years. The overall IR for TV ranged from 0 to 27 per 10,000 person-years. The highest age-specific testing positivity rate and IR rate for BV and VVC occurred in women aged 20–34 years and 20–34 years. For TV, female IR for BV, VVC, and TV remained stable during the study.

**Discussion:**

These unique regional data provide insight for the development of appropriate age-specific clinical testing criteria according to relative risk of acquisition of each vaginitis agent.

## Introduction

Vaginitis may cause inflammation of the vagina that is characterized by an inflammatory response attributable to changes in the local microbial milieu (1, 2), but this may not occur in all cases of vaginitis. Vaginal discharge, the predominant symptom in vaginitis, has a characteristic odor and color, while other symptoms include erythema, pruritus, dysuria, and dyspareunia. Vaginitis is most commonly caused by bacterial vaginosis (BV), vulvovaginal candidiasis (VVC), and *Trichomonas vaginalis* (TV), with these being the most prevalent etiologies (2–6). These infections occur primarily in women, although a small proportion of men may also become infected. BV is the most common vaginal disorder globally in both pregnant and non-pregnant women. Recent microbiome studies have shown the characteristic microbial ecosystem dysbiosis associated with the condition (7–10). Specifically, lactobacilli predominance decreases with an overgrowth of facultative aerobic and anaerobic organisms such as *Gardnerella vaginalis*, *Atopobium vaginae, Mycoplasma hominis,* and *Prevotella* and *Mobiluncus* species. BV is associated with a higher acquisition of other STIs, particularly *Chlamydia trachomatis* and *Neisseria gonorrhoae* (11, 12). Overgrowth of *Candida albicans* causes most cases of VVC, but other *Candida* species or yeasts may also cause vaginitis. Although women may have a whitish curd-like discharge, other symptoms are non-specific. Vaginitis caused by the flagellated parasite *Trichomonas vaginalis* presents in a majority of women with a foul-smelling discharge, genital itching, and dysuria.

As the symptomatology of infectious vaginitis has considerable overlap, improved molecular diagnostics are expected to enhance diagnostic accuracy and precise management (13). BV diagnosis is based on the Amsel criteria or microscopic examination of vaginal fluid and assessment of the Nugent score, which quantifies pathogenic and *Lactobacillus* bacteria (14). New BV nucleic acid amplification tests (NATs) have recently become commercially available (15, 16). Traditional laboratory diagnosis of VVC relies on the microscopic examination of vaginal fluid and culture (e.g., recurrent infection or treatment failure), but new NATs with improved sensitivity have become commercially available (15, 16). Implementation of TV NAT testing in 2016 improved the sensitivity of detection in our region compared to either microscopy or lateral flow assay detection (17).

Vaginitis testing represents one of the highest tests consistently ordered in outpatient community-based practice settings in our region. It was of interest to study the epidemiology of infectious vaginitis in a contemporary population-based cohort in the era of molecular testing because of limited prior publications in this important field. We hypothesized that rates of vaginitis in our large Canadian healthcare zone would be highest for BV > yeast>TV despite the universal use of NAT for the detection of the later pathogen by the laboratory.

## Materials and methods

### Patient selection

A retrospective cohort study was conducted on a large Canadian health region population who underwent testing for vaginitis between March 2015 and March 2018. Population estimates and census data for age-standardized estimates for 2016 were obtained from the Calgary Economic Development Commission and Statistics Canada, respectively.

### Laboratory setting

All laboratory testing was conducted by the centralized regional clinical microbiology laboratory, Alberta Precision Laboratories, Calgary Zone (APL-Calgary) in Calgary, Alberta. This facility serves an urban and rural population in Calgary and surrounding hamlets of ~1.6 million individuals including hospitalized, ambulatory, and long-term care patients.

### Data source

Digital test records of all testing for BV, VVC, and TV that were performed by the centralized regional clinical microbiology laboratory, Alberta Precision Laboratories, South Zone (APL-SZ) in Calgary, Alberta, were linked to demographic information including sex and age using the provincial healthcare number. Our dataset includes incident and prevalent cases because regardless of the pathogen, vaginitis can often be asymptomatic.

### Laboratory methods

In brief, regional physicians collected a single, red-topped liquid Amies swab for testing for both BV and VVC. A second vaginal swab was collected using the APTIMA™ Multitest kit for testing for *Chlamydia trachomatis/Neisseria gonorrhoeae* and/or *Trichomonas vaginalis*. All vaginal swabs were transported to the APL-Calgary microbiology laboratory within 4–8 h of collection. Vaginal swabs were immediately processed by preparing a thin-layered smear on a clean microscopic slide, which was then heat-fixed prior to Gram-staining. BV was diagnosed by highly trained medical laboratory technologists (MLTs) using the Nugent’s score (14), and the slide was also examined for the presence of moderate to considerable amounts of yeast. Vaginal swabs were not routinely cultured to recover yeast unless the physician provided a history of recurrent infection or treatment failure. All TV testing was done by the local laboratory using one of two available commercial nucleic acid amplification tests (NATS); (1) the APTIMA™ Combo (*Chlamydia trachomatis*/*Neisseria gonorrhoeae/Trichomonas vaginalis*) or (2) the APTIMA™ TV test(s) depending on the physician order. All NAT assays were performed on a Panther system (Hologic, Marlborough, MA, USA) strictly according to the manufacturer’s instructions.

### Statistical analysis

The incidence and age-standardized rates of BV, VVC, and TV were calculated using previously established methods (18). We calculated annual incidence rates by sex and age groups using laboratory and population estimates or census data. Age-specific incidence rates in Southern Alberta were calculated for each year according to 5-year groups from 0 to 75+ years. Study outcomes were established prior to data collection. Testing proportions were derived from APL test numbers compared to the population (according to published Calgary census data from 2016), and positivity proportions were calculated based on test numbers annually.

Incidence rate ratios (IRRs) and incidence rate differences (IRDs) were calculated to determine differences in testing and positivity rates over the study period. Figures of sex and age-standardized rates were constructed using the 2016 Calgary census data as a representative of the total data. All hypotheses were two-sided with *α* significance of 0.05. Clinical data were analyzed using STATA 15 software (College Station., Texas).

### Ethics

This study was approved by the Conjoint Health Research Ethics Board (CHREB), Alberta Health Services, and the University of Calgary (REB13-1126).

## Results

### Baseline cohort characteristics

Enrolled patients included both females and males ranging in age from birth to the oldest age group (≥75 years) who had a diagnostic test done for either BV/yeast and/or TV during the study period. The annual mean population in 2016 was 1,411,660 individuals (50.3% female), with the at-risk population being similar across age groups, ranging from approximately 40,000–60,000 individuals for each group. However, for those aged 65–69 and 70–74, the at-risk population was approximately 30,000 and 20,000, respectively. Table 1 outlines the distribution of ordered tests according to sex. A total of 293,962 enrolled patients were evaluated for both BV and yeast infections. The majority of patients were female (293,853, 99.96%), while only 106 tests (0.04%) were done on men. A total of 340,086 enrolled patients were evaluated for TV. The majority of patients were women (339,953, 99.96%), while only 130 tests (0.4%) were done on men. The sex was unknown for three patients.

**Table 1:** Distribution of testing for BV/Yeast and/or TV by gender.

Age	Females				Males				Grand totals	
Groups	BV/Yeast tests	%BV/Yeast tests	TV tests	%TV tests	BV/Yeast tests	%BV/Yeast tests	TV tests	%TV tests	Total number of tests	%Total tests
0–4	39	0.013	39	0.011	1	<0.0001	1	<0.001	80	0.01
5–9	44	0.015	44	0.012	0	0	0	0	88	0.01
10–14	499	0.17	634	0.19	0	0	0	0	1,133	0.18
15–19	14,376	4.90	22,269	6.51	14	0.005	23	0.007	36,682	5.76
20–24	40,065	13.63	54,404	15.90	32	0.011	47	0.14	94,548	14.86
25–29	54,341	18.49	64,761	18.92	23	0.008	23	0.007	119,148	18.72
30–34	53,149	18.08	59,389	17.35	5	0.002	5	0.001	112,548	17.69
35–39	39,669	13.49	43,637	12.75	7	0.002	7	0.002	83,320	13.10
40–44	28,412	9.67	30,720	8.98	5	0.002	5	0.001	59,144	9.30
45–49	21,058	7.16	22,462	6.56	5	0.002	5	0.001	43,530	6.84
50–54	15,263	5.19	15,985	4.67	2	<0.0001	2	<0.001	31,254	4.91
55–59	11,112	3.78	11,549	3.37	3	0.001	3	<0.001	22,667	3.56
60–64	7,376	2.51	7,612	2.22	3	0.001	3	<0.001	14,994	2.36
65–69	4,549	1.55	4,652	1.36	2	<0.0001	2	<0.001	9,205	1.45
70–74	1742	0.59	1749	0.51	2	<0.0001	2	<0.001	3,495	0.56
75+	2,159	0.73	2,180	0.64	2	<0.0001	2	<0.001	4,345	0.68
Grand total	293,853	99.96	342,086	99.94	106	0.040	130	0.040	636,181	100

**Figure 1:**
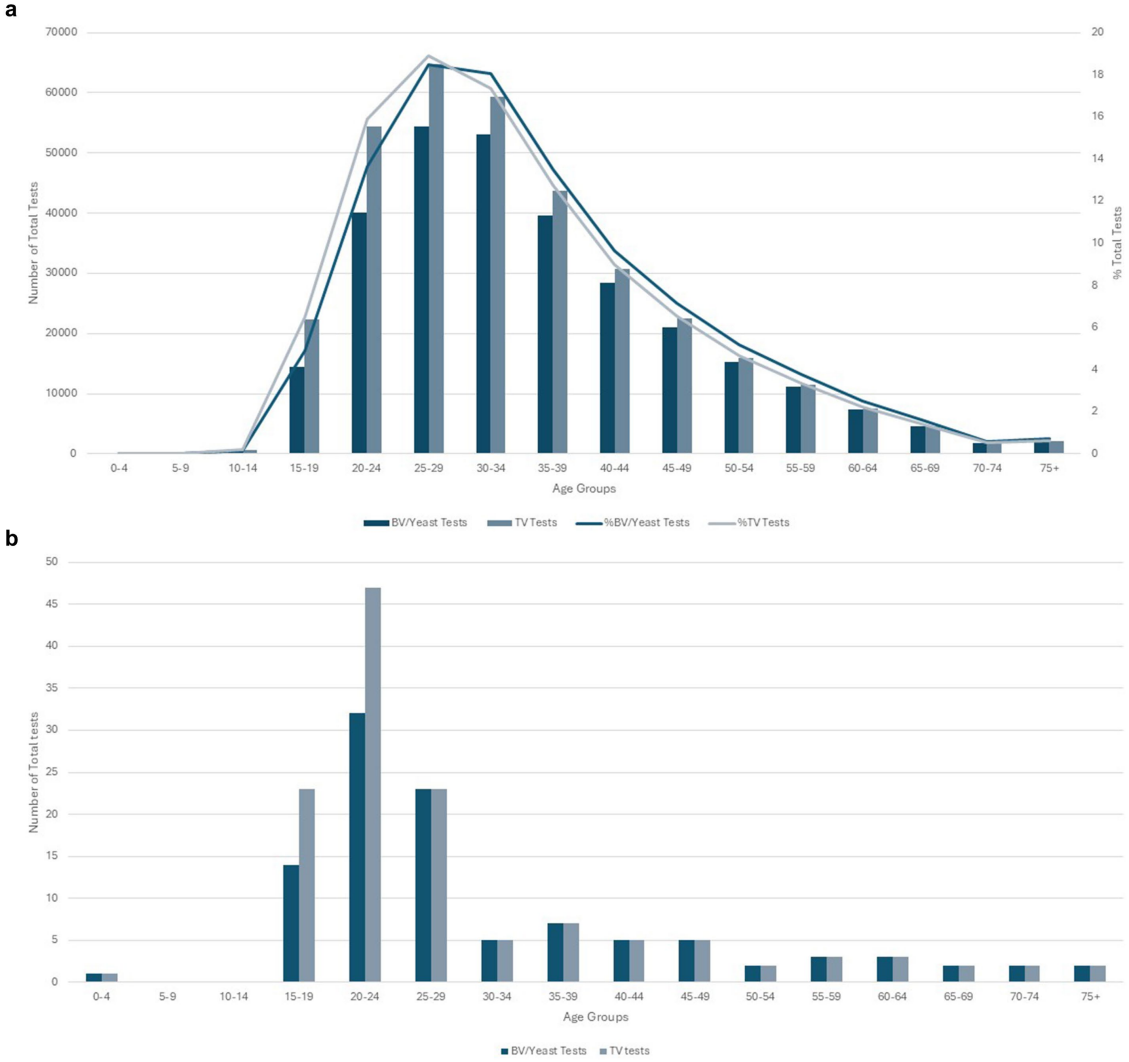
**(A)** Number of testes done for delection of BV/Yeast and *Trichomonas vaginalis* in females by age group (2016–2018). **(B)** Number of testes done for delection of BV/Yeast and *Trichomonas vaginalis* in males by age group (2016–2018).

The age-specific distribution of patients evaluated for BV/yeast and/or TV is shown for women in Figure 1A and for men in Figure 1B. The peak ages for infectious vaginitis testing in female patients for all three pathogens were between 20 and ≤ 35 years. The peak ages for testing in male patients for all three pathogens were ≥ 15 years to ≤29 years. Fewer tests were done on both sexes at the extremes of age including children ≤14 years of age and older adults ≥75 years of age.

### Testing and positivity rates

**Figure 2:**
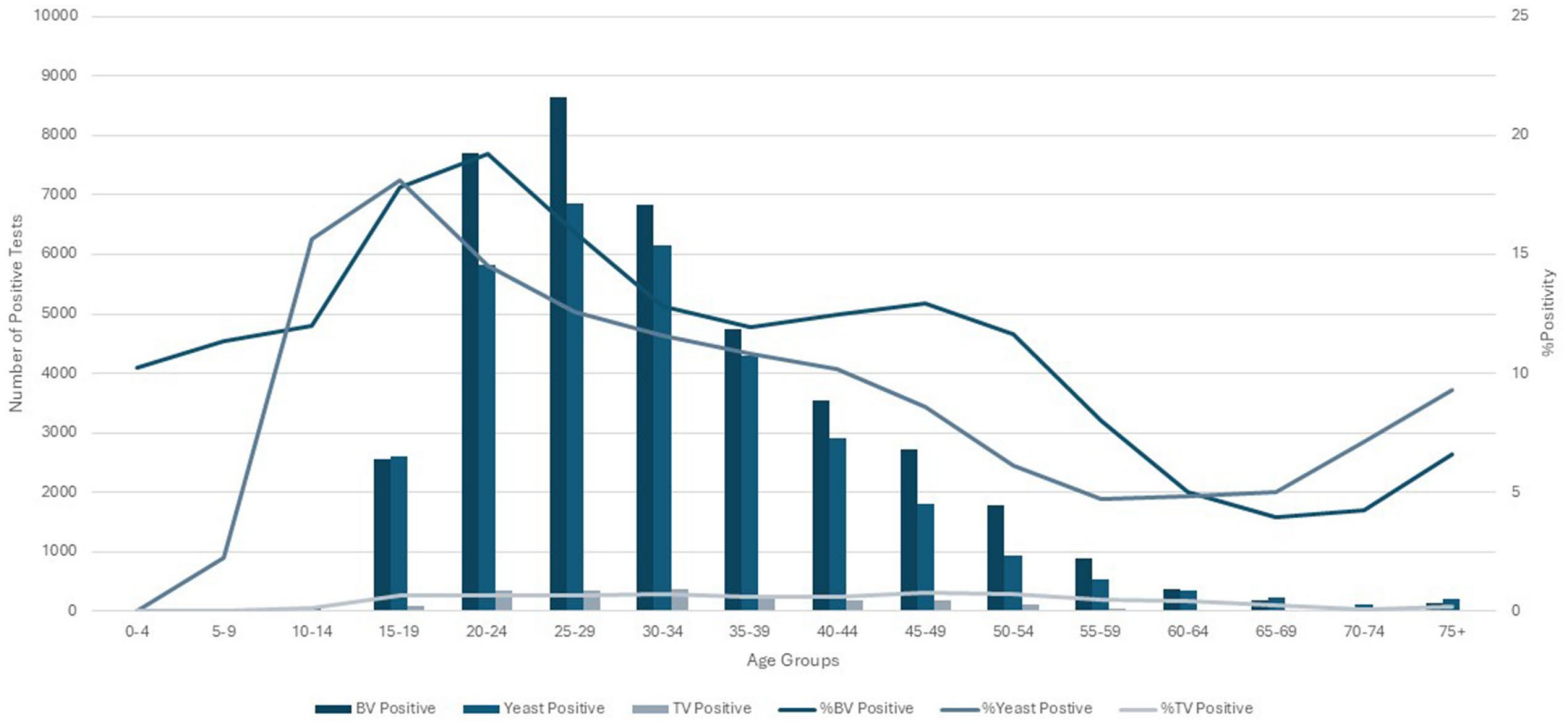
Female age specific testing and positivity rates for bacterial vaginosis, yeast and *Trichomonas vaginalis* test (2016–2018).

Increased testing positivity rates for female sex and younger age groups were associated with increased risk of BV, VVC, and TV infections (Figure 2). A total of 40,259 cases of BV were confirmed among 293,853 tests for an overall positivity rate of 14% (range = <4% for children 0–4 years up to 19% for those aged 20–24 years). Overall BV positivity rates were highest among those aged ≥15 to ≤29 years. A total of 32,894 cases of VVC were confirmed among 293,853 tests for an overall positivity rate of 11% (range = 0% for children 0–4 years and up to 18% for those aged 15–19 years). Overall VVC positivity rates were highest among those aged ≥10 up to ≤24 years. A total of 2018 cases of TV were confirmed among 342,086 tests for an overall positivity rate of 1% (range = 0% for children ≤14 years and up to 1% for those aged ≥15 but ≤54 years).

As shown in Table 1, the number of overall tests done on male patients across all age groups was too low to calculate accurate overall or age-specific positivity rates for all three pathogens. Figure 1B shows the age-specific distribution of the 15 BV-positive tests and eight yeast-positive tests in male patients. Only one male patient (aged 40–44 years) evaluated positive for TV.

### Incidence rates and incidence rate ratios

The % positivity rates for BV, yeast infections, and TV during the study are shown in Figure 2. Cases of all types of infectious vaginitis in female children ≤14 years were extremely rare. The overall BV IR was 184 cases per 10,000 person-years (95% CI = 157, 211). Female patients aged 20–24 and 25–29 years had the highest BV IRs of 585 and 609 cases per 10,000 person-years, respectively. The lowest adult BV IR of 10 cases per 10,000 person-years occurred in older female adults ≥75 years. Female patients ≥15 years had an IRR for BV of 61 and an IRD of 599 cases per 10,000 person-years. The overall yeast IR was 151 cases per 10,000 person-years (95% CI = 127, 175). Female patients aged 20–24 and 20–29 years also had the highest yeast IRs of 445 and 403 cases per 10,000 person-years, respectively. The lowest IR of 14 cases per 10,000 person-years of yeast occurred in older female adults ≥75 years. Female patients ≥15 years had an IRR for yeast infections of 32 and an IRD of 431 cases per 10,000 person-years. TV had a much lower overall incidence rate across all female age groups compared to either BV or yeast infections. The overall TV IR was 9 cases per 10,000 person-years (95% CI = 3, 15). No cases of TV were diagnosed in children and older female adults over 70 years of age. The highest TV IR of 27 cases per 10,000 person-years occurred in female patients aged 20–24 years, but similar IRs were found for women up to 34 years. The lowest IR rate of 2 cases per 10,000 person-years occurred in female patients aged 65–69 years. Female patients ≥15 years had an incidence rate ratio (IRR) for TV of 9 and an incidence rate difference (IRD) of 24 cases per 10,000 person-years.

**Figure 3:**
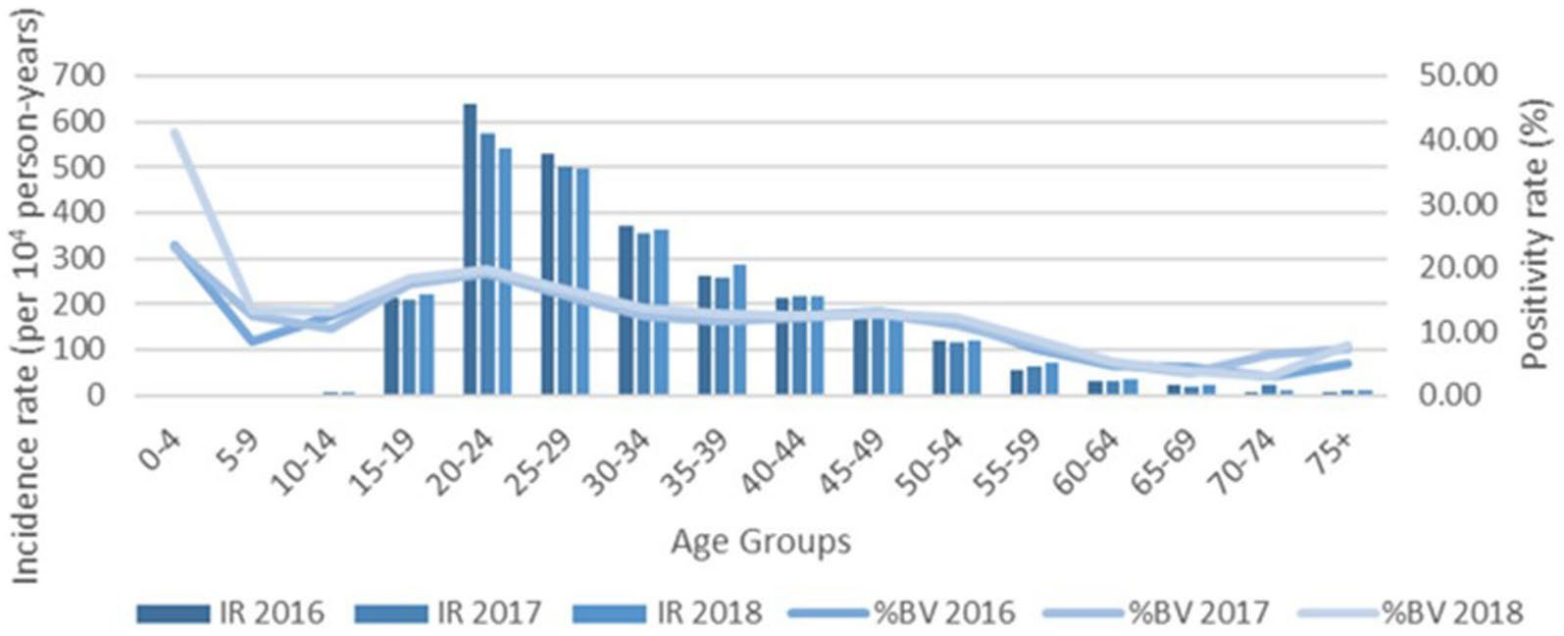
Female age specific testing and positivity rates for bacterial vaginosis by year.

**Figure 4:**
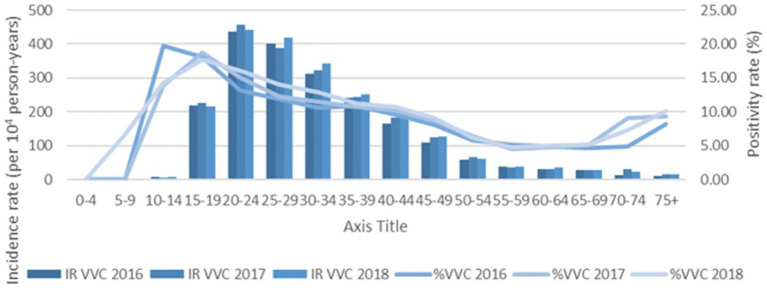
Female age specific testing and positivity rates for yeast by year.

**Figure 5:**
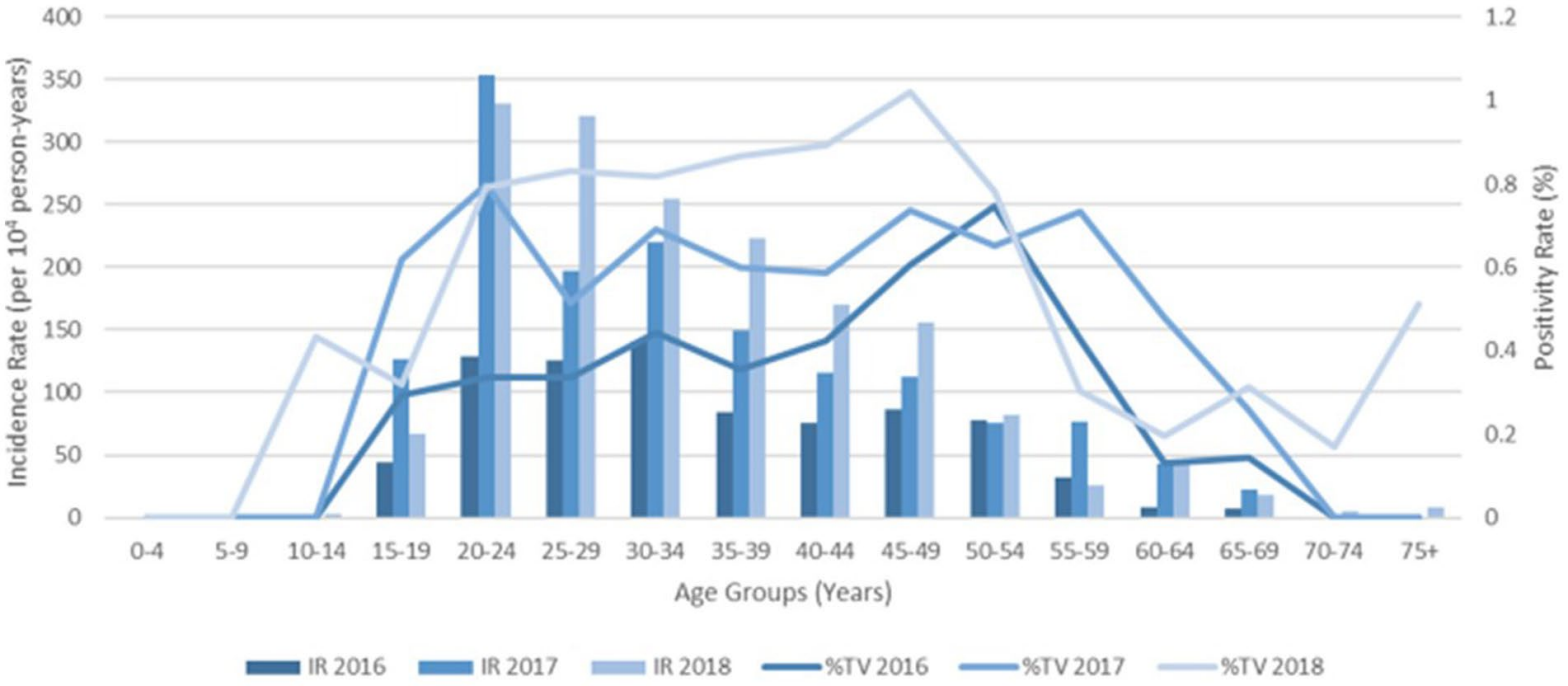
Female age specific testing and positivity rates for *Trichomonas vaginalis* by year.

The IRs for BV and yeast infections remain stable during the study as shown according to pathogens in Figures 3, 4. The TV rates were lower in female patients aged 15–39 years in 2016 but remained stable for the remainder of the study period (Figure 5).

## Discussion

This multi-year population-based study of infectious vaginitis is the largest population-based epidemiologic assessment reported to date in a large Canadian integrated healthcare region served by a single laboratory provider. Limited epidemiologic data of BV and yeast infection rates have been reported from the general populations of either North American or European jurisdictions (11, 19, 20). These data are necessary to inform policy regarding testing requirements as well as appropriate laboratory test utilization for specific vaginitis pathogens. As expected, the majority of tests were conducted on female patients, with only 0.04% of tests ordered for male patients. However, there was also a discrepancy in pathogen-specific testing among female patients across all age groups. Although healthcare providers evaluated 1.2X more female patients for TV than tests for the other pathogens, the overall positivity rate for TV (1%) was considerably lower than either BV (14%) or yeast (11%). This practice may reflect the ease of requesting a TV NAT test when collecting a single-vaginal multi-test swab for also testing for both *Chlamydia trachomatis* and *Neisseria gonorrhoeae*. Although BV/yeast had much higher IRs in our region, physicians ordered more TV NAT tests; a total of 293,962 individuals were tested for BV/yeast infections, while 340,086 were tested for TV. This discrepancy occurred because a NAT TV test was ordered as a combination test that included both *C. trachomatis* and *N. gonorrhoeae* in women suspected of having cervicitis and vaginitis.

Female age-specific rates of BV and VVC were highest among women of reproductive age, but cases occurred among all age groups. Both testing rates and positive cases for BV and yeast infections were highest as expected in the 15–40 years age groups, but age-specific IRs were the highest for women between ages 20 and 29 years. The overall BV/yeast IR rates are, however, much higher than those of TV and other STIs in our region. This makes BV/yeast the most common cause of vaginitis in women in our general regional population, particularly those of reproductive years who are sexually active and symptomatic. BV/yeast IRs also exceeded those of other major STIs in our general population. Somayaji et al. (21) found the overall IR for chlamydia ranged from 18.81 to 25.63 cases per 10,000 person-years, and the overall IR for gonorrhea ranged from 0.92 to 1.86 cases per 10,000 person-years.

The infection rates for TV in this study agree with the low prevalence (0.5%) previously reported in our general population using non-molecular methods of detection (17). According to this study, the TV rate (1%) in our general population increased with molecular detection, but it remains considerably lower than previously published data for young adults in the United States (3.2%) (22, 23). Our TV prevalence rate (1%) is also lower than that recently found among women (2.8%) attending the Edmonton Provincial STI clinic but is higher than the rate found among men (0.2%) (24). TV vaginitis in women was found to be associated with symptoms and non-white ethnicity (24). Limited epidemiologic data on TV infection rates have been reported from other areas. A low TV positivity rate was found in three patient cohorts from general practitioners, STI clinics, and a national population-based chlamydia screening study in the Netherlands (25). Studies from China document high rates of TV infection in women of reproductive age presenting with vaginitis symptoms. The global TV prevalence among high-risk populations attending STI clinics is up to 20% (26). Geographic variation in disease prevalence has also been documented for other types of bacterial STIs (27).

Limitations of our study on infectious vaginitis rates include the sample population and the tests used for diagnosing BV and yeast infections. Microscopic examination for these agents may have a lower sensitivity than using a NAT method (15, 28), and IR rates should be reassessed after the implementation of molecular testing. A prospective study of male patients (with gender information) would be ideal to determine accurate epidemiologic rates in that cohort. Although the clinical history was not available, ordering physicians use our laboratory guidelines along with their clinical assessment to establish a reasonable pre-test probability before evaluation. The decreased positivity rates for TV in the first year of the study may reflect the transition from using a lateral flow assay to NAT in the preceding months. Physicians likely needed time to become familiar with the new collection and testing protocols after the change in testing methods. However, TV NAT was the method used throughout the entire study, thus mitigating the impact of a changing study platform on infection prevalence. It is also possible that patients had multiple positive tests for the same infection or recurrent infections, but the laboratory monitors for duplicate test orders within a 3-week period.

Population-level modeling, including the monitoring of IRs in the general population, will assist in developing age-specific testing criteria for core groups with the highest infection rates, aiming to prevent the spread of infectious vaginitis and associated STIs in the region. Our data allow the use of age-specific criteria for directing healthcare providers to assess female patients more appropriately for vaginitis-specific pathogens. Reduced vaginitis testing may be appropriate for female patients aged ≤14 years and older adults, in the absence of risk factors or symptoms, particularly for TV, where the pre-test probability is exceptionally low. In our region, the majority of vaginitis testing (99.5%) in female patients ≤14 years is due to early onset of sexual activity rather than sexual assault. Physicians typically order TV testing when conducting STI tests (i.e., *C. trachomatis*/*N. gonorrhoeae*). These data also support the implementation of NAT for the simultaneous detection of vaginitis pathogens using a single-vaginal multi-test swab. The recent implementation of BV/yeast NAT testing in our region would also potentially allow reflex testing where first a BV/yeast test is done, and TV testing only proceeds if the former testing is negative. Additional population-based studies of vaginitis in other healthcare jurisdictions would provide insight into vaginitis testing guidelines for specific microbial agents. Potential linkages of infectious vaginitis to the acquisition of other STIs also warrant further study.

## Data Availability

Publicly available datasets were analyzed in this study. This data can be found here: data is available with permission from Alberta Precision Laboratories, Alberta Health Services.
